# Investigating the closure stress and crack initiation stress in fractured rocks using the student *t* distribution and Monte Carlo simulation method

**DOI:** 10.1371/journal.pone.0307804

**Published:** 2024-08-07

**Authors:** Hanjie Lin, Yue Qiang, Li Li, Hongjian Li, Siyu Liang

**Affiliations:** Department of Civil Engineering, Chongqing Three Gorges University, Wanzhou, Chongqing, China; Southwest Petroleum University, CHINA

## Abstract

Traditional method of determining closure and initiation stress of fractured rocks by analyzing the stress-strain curve has problems such as strong subjectivity and large errors. This study utilized the rock closure stress values and onset stress values determined by three traditional methods, namely, axial strain method, fracture volume method and empirical value taking method, as the base database. The Student *t* distribution theory was used to obtain a confidence interval based on its overall distribution of values and to achieve a combination of the advantages of multiple methods. Within confidence interval, the Monte Carlo stochastic simulation was used to determine the convergence interval of the second stage to further improve the accuracy. Finally, mean value of the randomly sampled values after reaching the convergence stage was taken as the probability value of rock closure and crack initiation stress. The results showed that the 3 traditional methods for calculating rock closure and initiation stresses are significantly different. In contrast, the proposed method biases more towards multi-numerical distribution intervals and also considers the preference effects of different calculation methods. In addition, this method does not show any extreme values that deviate from the confidence intervals, and it has strong accuracy and stability compared to other methods.

## 1 Introduction

Rocks are complex structures in nature that are subject to physical weathering, water erosion, geological and tectonic movements. This leads to changes in the mechanical properties of rocks and adversely affects engineering activities. Therefore, calculating the strength eigenvalues of rocks by analyzing their stress-strain curves is very important for engineering practice activities [1, 2].

A large number of indoor experimental studies have shown that the deformation and damage process of rocks mainly consists of five stages: fracture compression stage, elastic deformation stage, fracture stable rupture stage, fracture accelerated rupture stage, and damage stage after exceeding the peak. They correspond to the strength characteristic values of closure stress, crack initiation stress, damage stress, peak stress, and residual stress, respectively [3–5]. It is obvious that the stage division of deformation damage of rocks is beneficial to predict the deformation development trend. The eigenvalues of peak stress and damage stress can be determined by analyzing the variation of stress-strain curves [5]. In contrast, the closure stress and crack initiation stress cannot be obtained by simply analyzing the stress-strain curve. Therefore, in comparison, the determination of the closure stress and crack initiation stress is more difficult. Therefore, the determination of the closure and crack initiation stresses is more difficult.

Currently, for the determination of closed stress and crack incident stress, scholars at home and abroad have proposed some novel methods through a large number of studies. For example, Eberhardt et al [6] proposed a technique based on strain gauge and acoustic emission methods for the determination of initiation and destructive stresses in rocks. However, the acoustic emission method is susceptible to large errors due to factors such as rock grain structure, material composition and external noise. Therefore, the method is not suitable for the determination of crack closure stresses in rocks. Peng et al [7] made a supplement to the study of Eberhardt et al. and obtained the crack closure stress by comparing the axial strain response analysis between the pre-loading and crack instability development stages of the rock. Cai et al [8] presented empirical formulas for fracture emergence thresholds and fracture damage thresholds of nodular, massive and blocky rock bodies. These formulas further complement the calculation of crack initiation stress and damage stress for different types of rocks. Han et al [9] analyzed the expansion of three-dimensional cracks inside the rock with different morphologies and the sensitivity effect of the length of crack ends to the angle of precession under dynamic compression conditions. From the above, it can be seen that the different rock types cause different crack morphologies. Therefore, Zhou et al [10] summarized the characteristics of different methods (crack strain model calculation method, acoustic emission sampling method, volumetric strain curve observation method and moving point regression method) when determining the initiation crack stress and damage stress. Data from uniaxial or triaxial compression tests are often discontinuous due to the internal structure of the rock. Obviously, combining several methods can achieve higher accuracy. Experiments have demonstrated the existence of an approximate linearity between the closure stress and crack initiation stress stages during compressive rock damage. Therefore, most of the traditional analytical-mechanical type methods, such as the crack volume strain method and the axial strain method [5, 11], are considered to be reliable methods for determining the rock closure stress and crack initiation stress.

In summary, combining multiple methods can improve the accuracy of the judgment because each method has a different focus. From the validation data of this paper (Table 1), it can be seen that the values of the empirical method are intermediate between the values of the axial strain method and the crack volume strain method. In addition, the results obtained by the axial strain method and the crack volume strain method are based on a large number of physical tests. Therefore, the physical test data at the two ends of the interval have a higher reliability compared to the empirical method. The traditional normal distribution has smaller values at both ends and larger peaks. However, Student *t* distribution has stronger heavy-tailed than the standard normal distribution, which can improve the above shortcomings of the traditional normal distribution [12]. Heavy-tailed makes the tails of the student *t*-distribution more likely to have lower peaks than the standard normal distribution, and the student t-distribution is also highly robust [13]. Stronger robustness will allow models to show better performance even when subjected to external disturbances and large data deviations. Monte Carlo algorithms are widely used in various fields such as industrial engineering [14, 15], operations research [16, 17], osmosis [18, 19], and finance [20] due to their simplicity, efficiency, and excellent stochasticity. With the development of computers, Monte Carlo simulation plays an important role in quantitative research [21]. The core idea of the Monte Carlo algorithm is to perform repetitive sampling or simulation so as to obtain the values we want by using the large number law and other inference methods [21]. Therefore, this study sampled a sufficient sample size through Monte Carlo random sampling to overcome the instability of models when they are not converging at an early stage.

**Table 1:** Test data from 5 sets of rock samples.

Specimen	Citation	The value	Axial strain method (MPa)	Crack volume strain method (MPa)	Empirical value method (MPa)	Lower confidence interval limit	Upper confidence interval limit
#1	BS06	Closure Stress	40	30	35.90	26.83	43.77
	Zhao et al. (2015)	Crack initiation stress	90	70	85.54	64.15	99.55
#2	BS16	Closure Stress	35	30	38.34	27.37	41.52
	Zhao et al. (2015)	Crack initiation stress	80	65	76.69	60.61	87.18
#3	BS18	Closure Stress	60	65	77.22	52.47	82.34
	Zhao et al. (2015)	Crack initiation stress	150	160	151.30	144.60	162.93
#4	Eberhardt et al. (1998)	Closure Stress	30	50	48.43	24.06	61.56
		Crack initiation stress	100	110	103.67	96.03	113.08
#5	Zhao et al. (2013)	Closure Stress	25	40	27.2	17.08	44.39
		Crack initiation stress	70	64	68.9	62.25	73.02

In this study, the distribution of values was first analyzed based on three conventional stress-strain curve analysis methods. Then, the Student t distribution [22, 23] was used to calculate the confidence intervals, thus absorbing the advantages of each method. Subsequently, the closure stresses and crack initiation stresses of the rock were calculated by using the Monte Carlo stochastic simulation algorithm [24, 25] within the interval. Finally, uniaxial compression test data from 5 sets of fractured rocks were input for model comparison and validation. In Section 2, this paper described the modeling process; In Section 3, this paper performed the example verification; In Section 4, this paper discussed the strengths of the model for this study; In Section 5, this paper summarized the conclusions of the full paper.

## 2 Materials and methods

### 2.1 Staging of fractured rocks before peak damage

**Fig 1:**
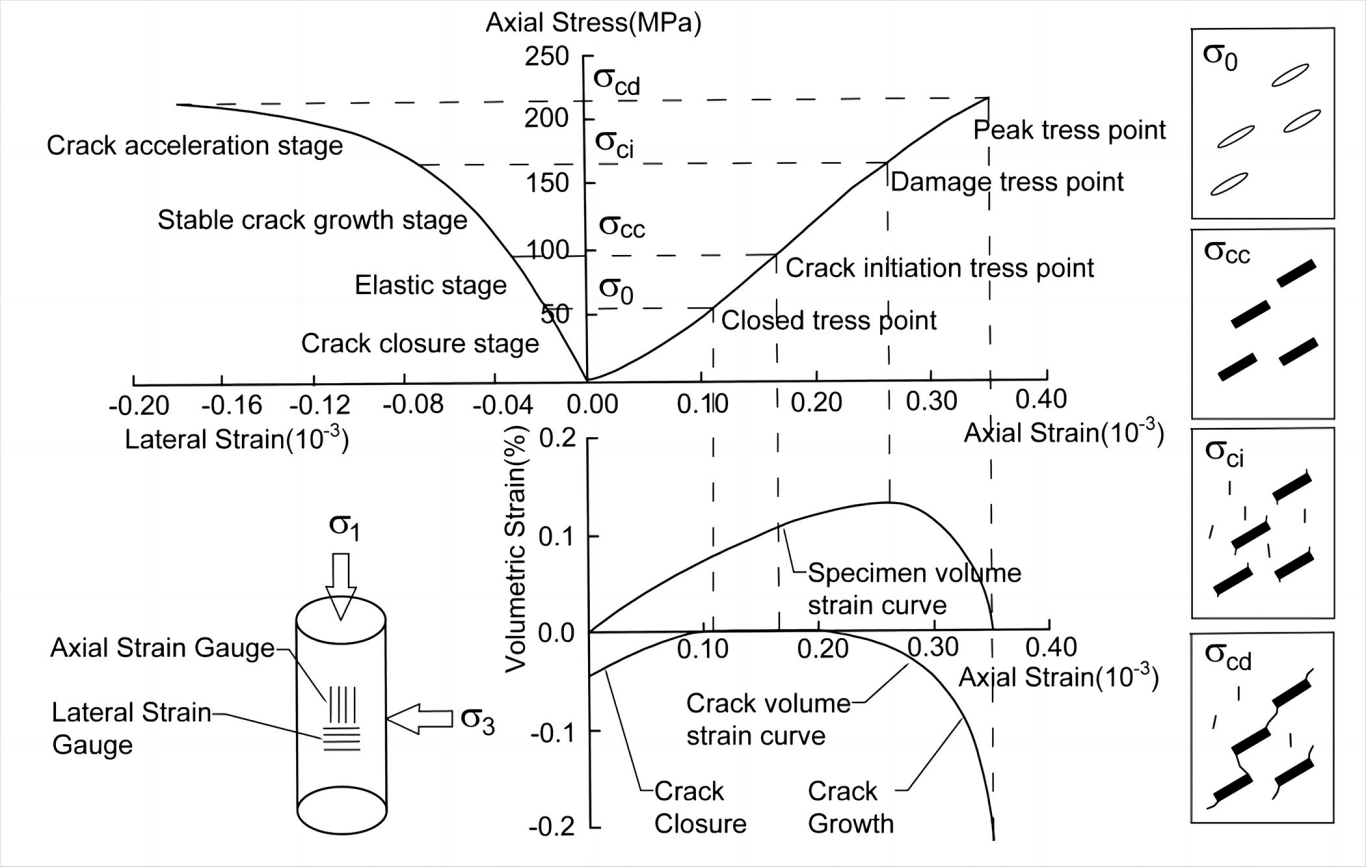
Staging of fractured rocks before peakdamage.

Extensive experimental studies have shown that there are 5 main stages of rock damage. Since the rock strength decreases sharply after peak damage, it is obviously more valuable to study the first 4 stages (Fig 1). In the first stage (Crack closure stage), the original small cracks in the rock are gradually compacted as the compressive strength increases. In the second stage (Elastic deformation stage), the fractured rock has been compacted at this time and can be considered as the elastic deformation of the rock itself. The stress-strain curve in this process grows linearly, and its starting and ending points are usually considered to be important in determining the closure stress of the cracked rock and the crack initiation stress. In the third stage (stable crack growth stage), the elastic deformation of the rock reaches the ultimate critical value, and new fine cracks begin to develop steadily. In the fourth stage (crack acceleration stage), the pressure continues to increase, leading to the development of small cracks into macroscopic large cracks. Even multiple cracks further develop into stress-concentrated crack zones until the rock specimen ruptures.

### 2.2 Axial strain method

**Fig 2:**
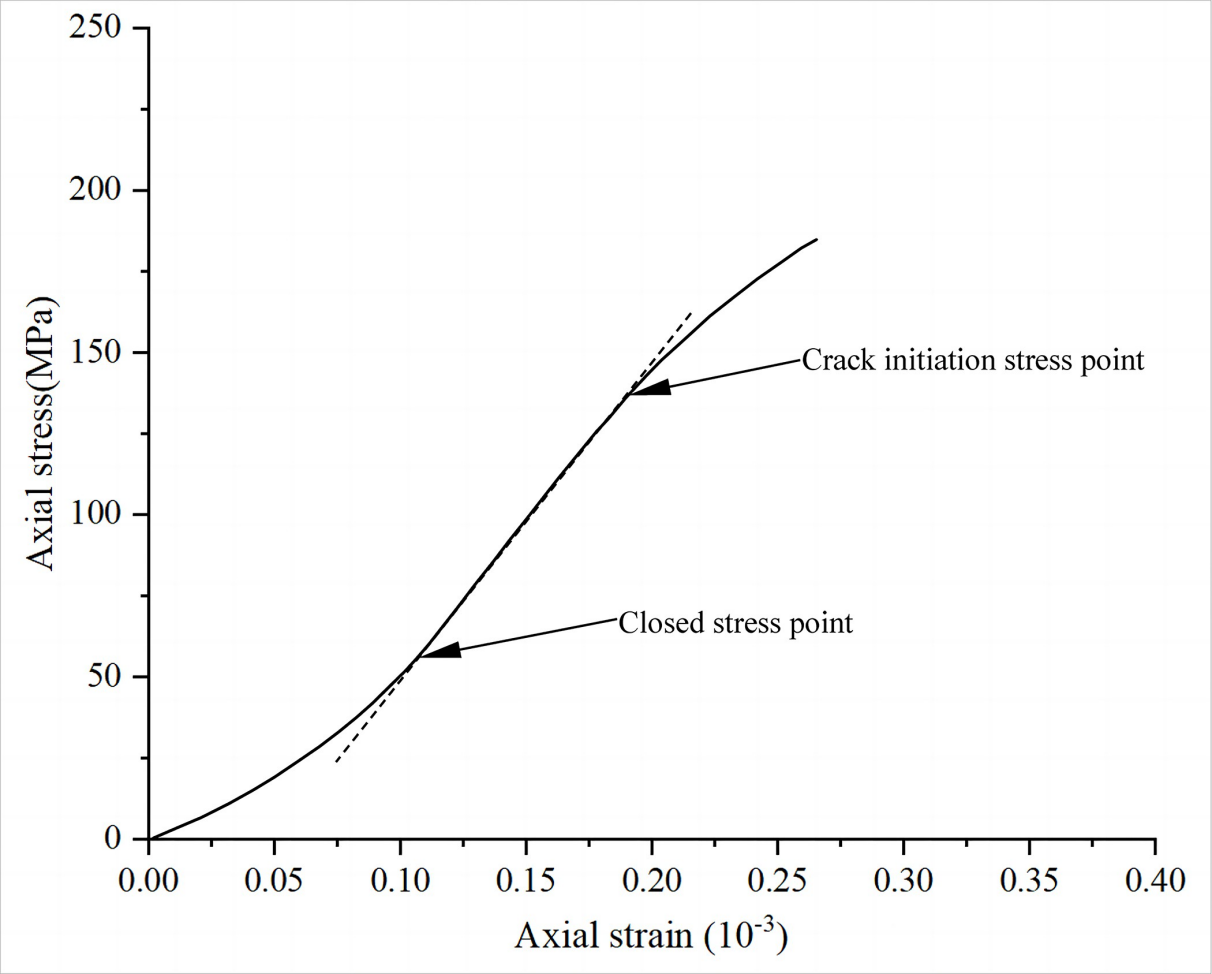
Schematic of the axial strain method.

In this section, this paper described the linear variation of the stress-strain curve of a rock during the elastic deformation phase. The closure stress of the rock at this point can be determined by directly observing the turning point from the crack closure phase to the elastic phase. Similarly, the crack initiation stress can be determined by directly observing the turning point from the elastic stage to the stable fracture stage (Fig 2). The advantage of this method is its simplicity. However, in the actual experiment, the turning point of each stage may not be clear due to different rock types. Therefore, it is easy to cause large errors in the results.

### 2.3 Crack volume strain method

The volumetric strain *ε*_*v*_ of the rock can be calculated from the axial stress-strain data and lateral stress-strain data of the fractured rock obtained from triaxial compression tests. As follows:

$$\varepsilon_{v}=\varepsilon_{1}+2\varepsilon_{3}$$ (1)

Where *ε*_1_ denotes axial strain, and *ε*_3_ denotes lateral strain.

Combined with the previous elaboration of the fracture closure phase and elastic phase of the rock, it is known that the volumetric strain *ε*_*v*_ is composed of two parts: the crack volume strain *ε*_*cv*_ and the elastic volume strain *ε*_*ev*_ of the fractured rock itself. According to the theory of elasticity, where the elastic volume strain *ε*_*ev*_ of the rock itself is as follows:

$$\varepsilon_{ev}=\frac{1-2v}{E}(\sigma_{1}+\sigma_{2}+\sigma_{3})$$ (2)

Where *σ*_1_ denotes the principal stress, *σ*_2_ and *σ*_3_ denote the surrounding pressure, *E* denotes the modulus of elasticity, and *v* denotes the Poisson’s ratio.

Similarly, the crack volume strain *ε*_*cv*_ of the rock can be expressed as:

$$\varepsilon_{cv}=\varepsilon_{v}-\frac{1-2v}{E}(\sigma_{1}+\sigma_{2}+\sigma_{3})$$ (3)

When performing uniaxial compression tests, Eq (3) simplifies to:

$$\varepsilon_{cv}=\varepsilon_{v}-\frac{1-2v}{E}\sigma_{1}$$ (4)

**Fig 3:**
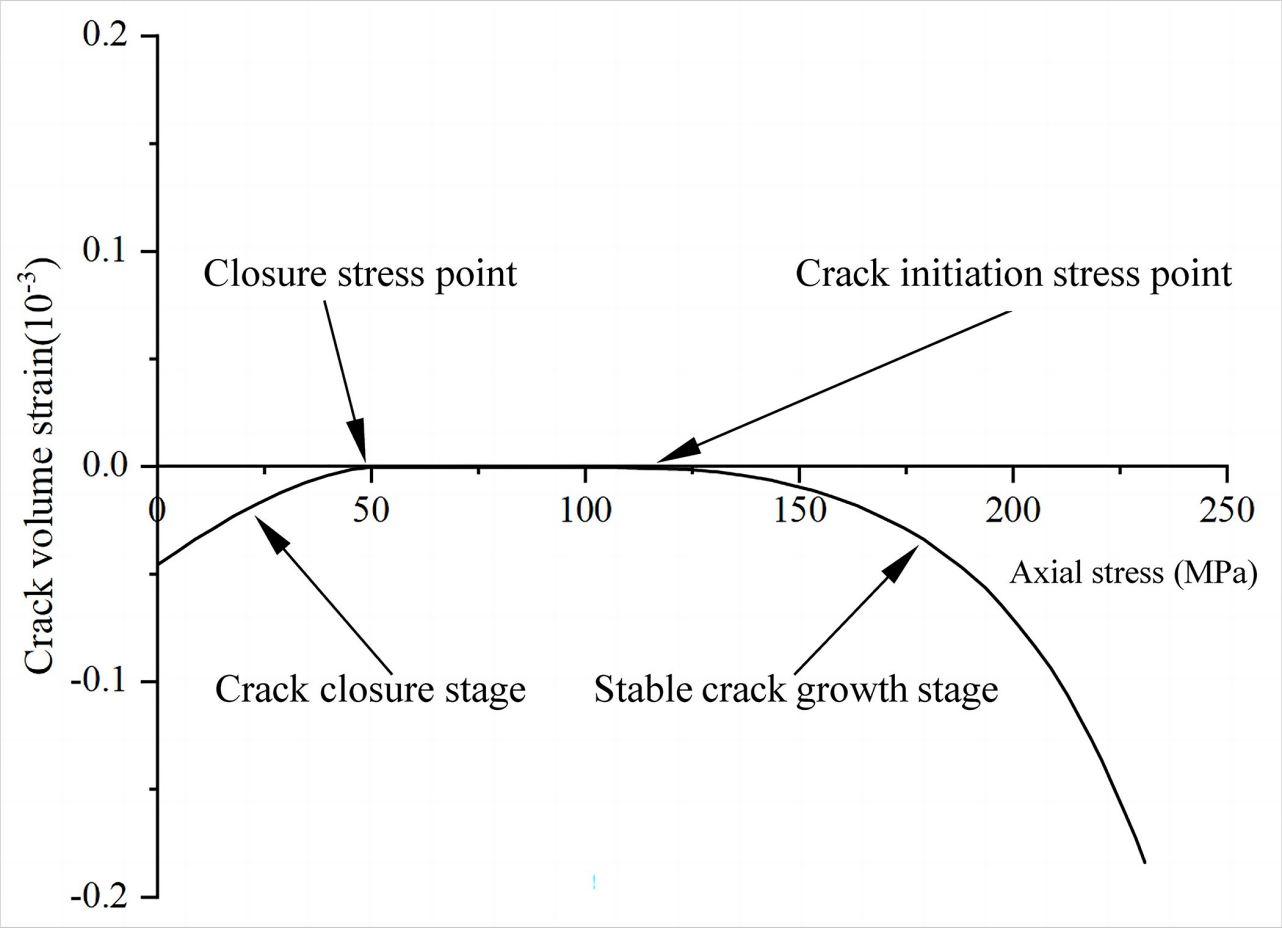
Schematic of the crack volume strain method.

The method relates the fracture volume strain to the axial stress and lateral enclosing pressure by Eq (3). When the crack volume strain *ε*_*cv*_ reaches 0, the stress is regarded as the closure stress. As the pressure increases, when the crack volume strain *ε*_*cv*_ deviates from 0, the stress is regarded as the crack initiation stress (Fig 3). Compared with the axial strain method by directly observing the fuzzy change nodes, this method quantifies the rock damage process and thus has higher accuracy. However, this method is influenced by the elastic modulus *E* and Poisson’s ratio *v*. The errors in the values of the parameters can easily lead the calculation results to deviate from the true values.

### 2.4 Empirical value method

**Fig 4:**
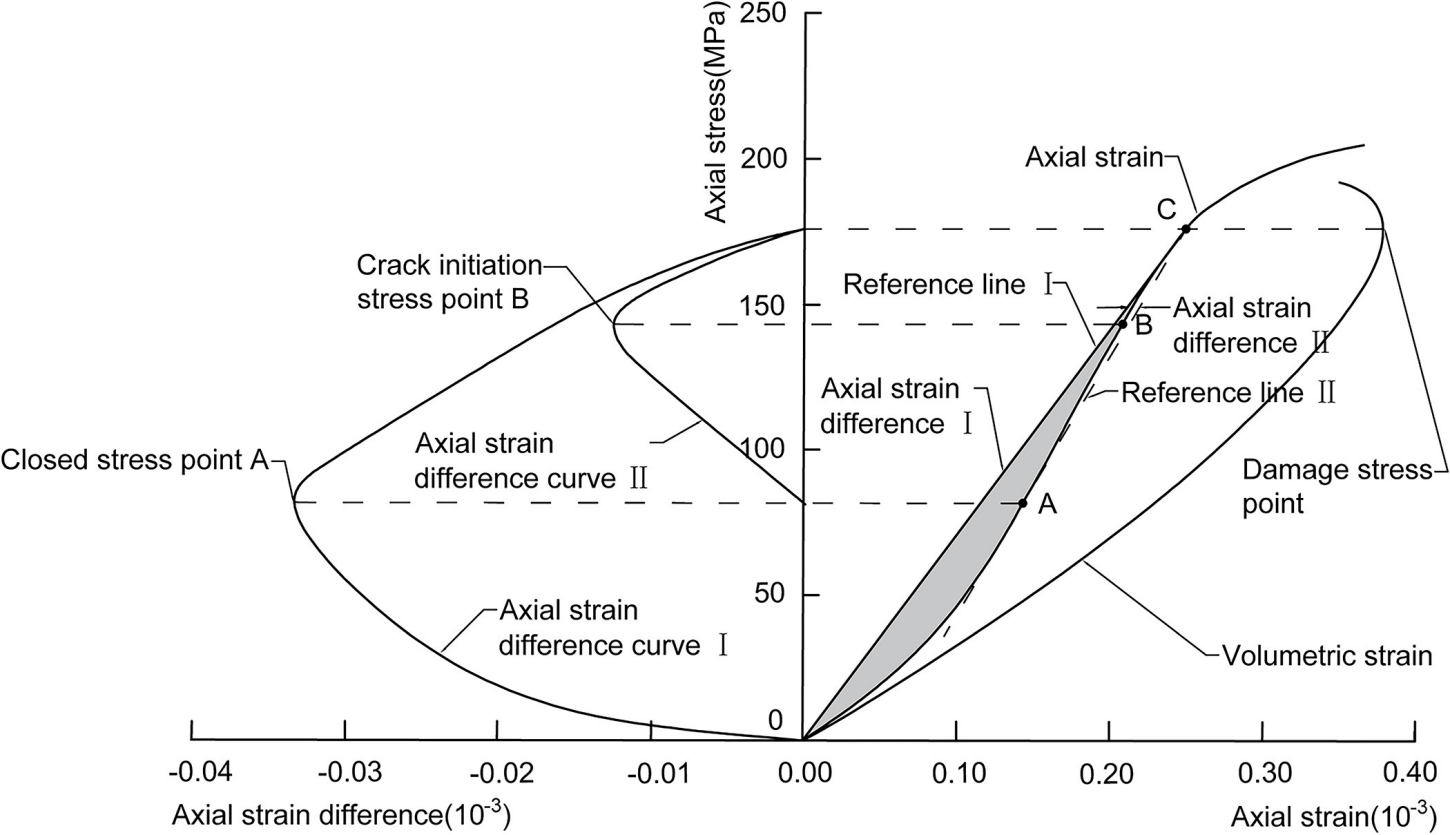
Schematic of the empirical value method.

Qiang [26] et al. proposed an empirical method to determine the rock closure stress and crack initiation stress based on the idea of Nicksiar et al [27]. The steps are as follows (Fig 4):

1. Determine the closed stress of the rock. Find the inversion point of the volumetric strain curve (destructive stress point); connect the projection point C of the destructive stress point on the axial strain curve with the origin O as an auxiliary line; calculate the axial strain difference between the axial strain stress curve and OC. The peak point of the axial stress of the strain difference fitting curve is the closed stress.
2. Determine the crack initiation stress of the rock. Make an auxiliary line connection between A and C, the projection point of the closed stress point on the axial strain curve; calculate the axial strain difference between the axial strain curve and AC. The axial stress at the peak point of the strain difference fitting curve is the crack initiation stress.

### 2.5 Probabilistic valuation method

We described the advantages and disadvantages of above 3 traditional methods for determining the closure stress and crack initiation stress in rocks. In order to fully combine the advantages of various methods, this study proposed a method based on probabilistic mathematical theory to determine the stress state of rocks. Considering the characteristics of the small sampling distribution, Student *t* distribution method with higher error tolerance was adopted. In addition, this paper used the sample standard deviation to determine the confidence intervals of the above three methods instead of the overall standard deviation. The Monte Carlo simulation method then continuously approximated the center by a large number of random samples to obtain a convergence interval with higher confidence within the confidence interval. Thus, the rock closure stress and crack initiation stress values with larger probability were calculated. The specific steps are as follows:

**2.5.1 Student *t* distribution principle.** For a normal distribution with a sufficiently large sample size, its standard deviation *σ* can be regarded as a constant value. However, the *σ* of the small sample has limitations and cannot be taken as a good estimate of the overall standard deviation. Therefore, considering the small sample sampling, this paper selected Student t-distribution to depict the standard deviation of the overall sample in order to minimize the error. Its statistical formula is as follows:

$$z=\frac{\bar{x}-\mu }{\sigma_{\bar{x}}}=\frac{\bar{x}-\mu }{\sigma /\sqrt{n}}$$ (5)

where $\bar{x}$ denotes the mean value of samples, *μ* denotes the localization parameter, *σ* denotes the scale parameter, and *n* denotes the number of samples.

**Fig 5:**
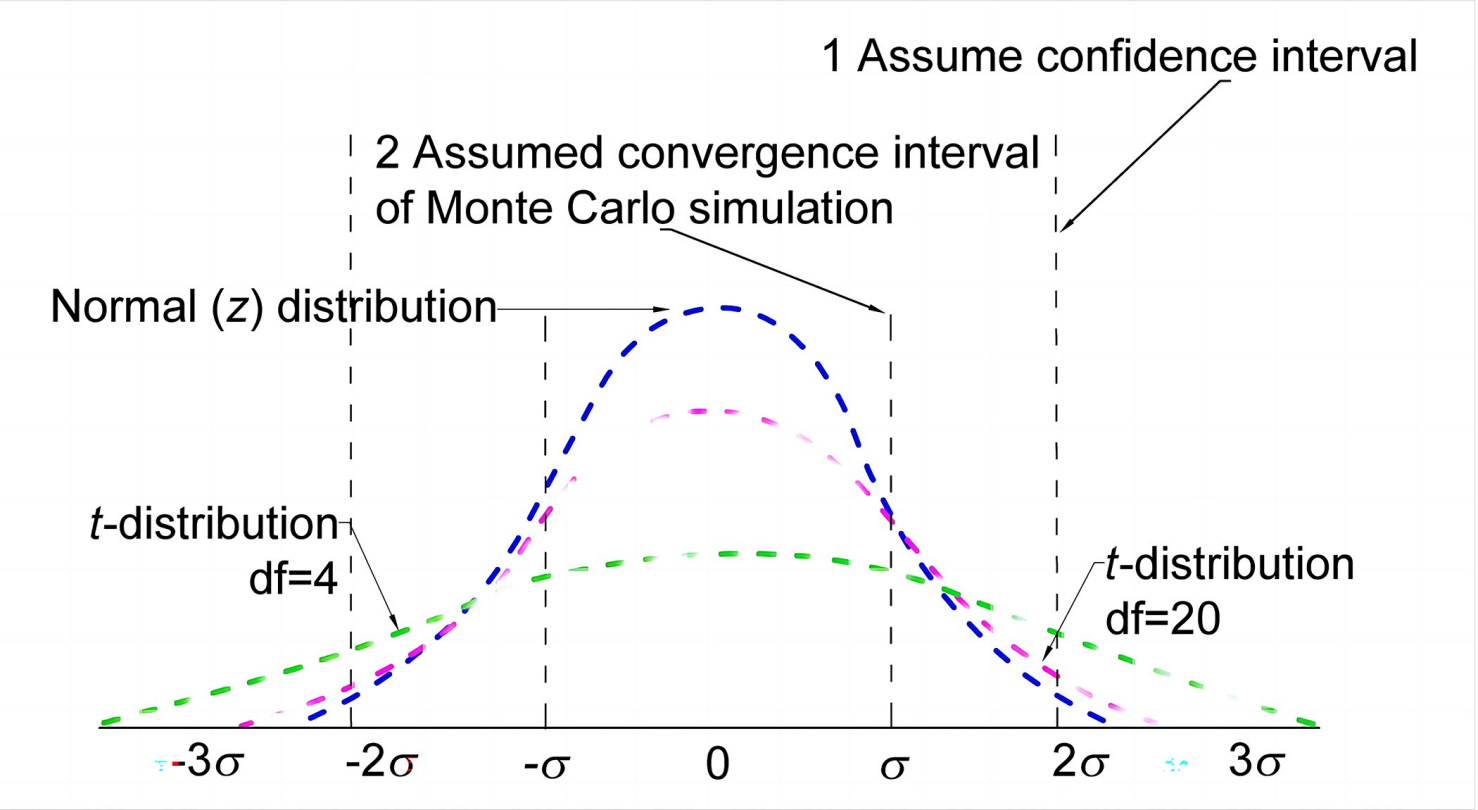
Schematic of the student *t* distribution method.

Compared to the normal distribution, the t-statistic contains two random variables, x-bar and s, and has greater variability. In addition, the variability of the student *t* distribution depends mainly on the sample size. Assuming a sample size of *n*, the degrees of freedom are expressed as df = n-1. When the degrees of freedom and sample size are small, the student *t* distribution is flatter than the normal distribution, and the error tolerance is higher. As the degrees of freedom increase and the sample size converges to the overall value, the distribution overlaps with the normal distribution (Fig 5).

Therefore, using the student *t* distribution method will yield stage 1 confidence intervals with higher error tolerances, as shown in the following equation.

$$\sigma \;unknow:\;\bar{x}\pm t_{\alpha /2}(\frac{s}{\sqrt{n}})$$ (6)

where *t*_*α*/2_ represents the t-value of the unilateral end area at *n*−1 degrees of freedom.

**Fig 6:**
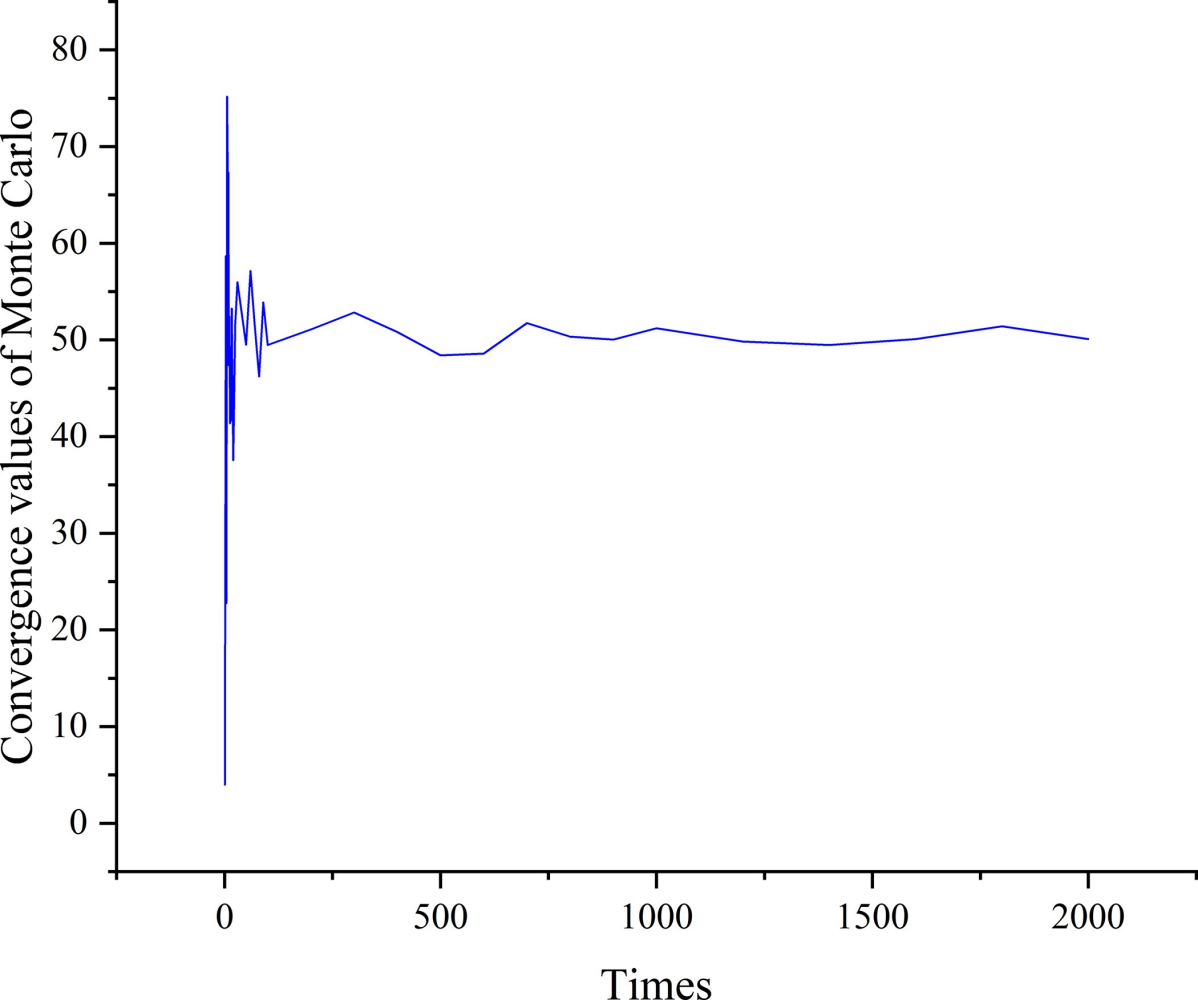
Schematic diagram of convergence of Monte Carlo method.

**2.5.2 Monte Carlo simulation principle.** The Monte Carlo algorithm, also known as the random sampling method, was introduced by Johnson in 1950. The method was initially designed to address a variety of random phenomena in the use of equipment. Its central idea is to solve complex practical problems by means of random sampling. Monte Carlo simulation algorithms approach the target by continuously approaching it with a large number of random samples, gradually converging from chaos to the target range. As the number of samples increases, the results of the Monte Carlo algorithm will gradually converge (Fig 6).

Common confidence intervals were calculated after the student t-distribution. Subsequently, by analyzing the convergence phenomenon of the Monte Carlo simulation method, the second-stage convergence interval with a higher confidence level was determined. From there, the rock closure stress and crack initiation stress values were determined within the interval. In order to achieve high accuracy, the following equations are proposed for intercepting the average value as the final value after reaching the convergence stage.

$$r_{i}=\frac{\sum_{n-n_{i}}^{n}\sigma_{l}+(\sigma_{u}-\sigma_{l})*rand(n,1)}{n-n_{i}}$$ (7)

where *r*_*i*_ denotes the final values of the closure stress and crack initiation stress for the *i*-th group of rock samples, *σ*_*l*_ and *σ*_*u*_ denote the lower and upper stress limits of the confidence interval, respectively, *n* denotes the total number of random samples taken, *n*_*i*_ denotes the number of samples taken when the convergence interval is reached; and *rand* represents random sampling.

**Fig 7:**
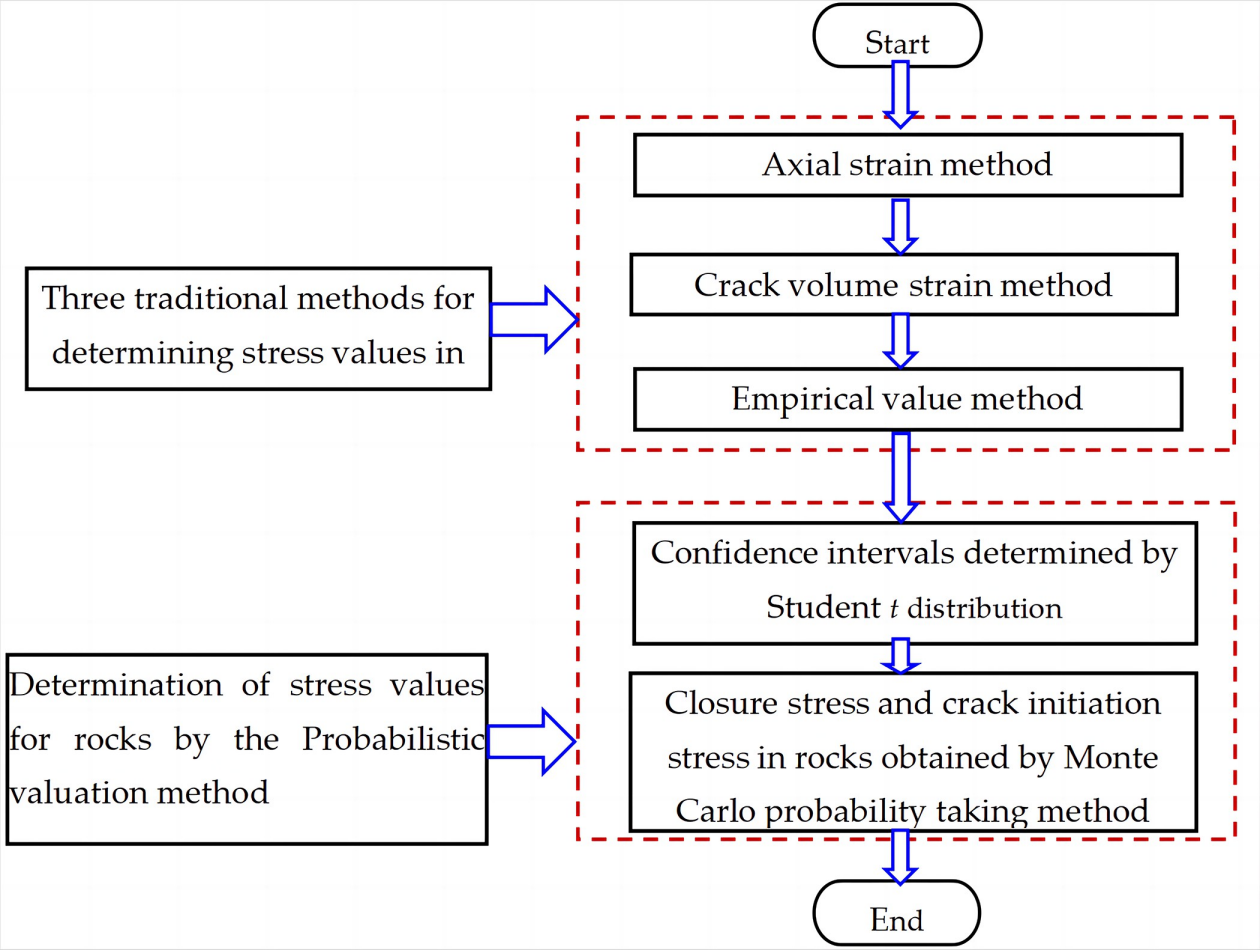
Model construction flow chart.

The model construction of the proposed method has been completed and the whole process is shown in Fig 7.

## 3 Results

### 3.1 Research area

Finally, uniaxial stress-strain data from 5 sets of rock samples tested by [6, 28, 29] (Table 1) were cited to verify the reliability of the method proposed in this study.

**Fig 8:**
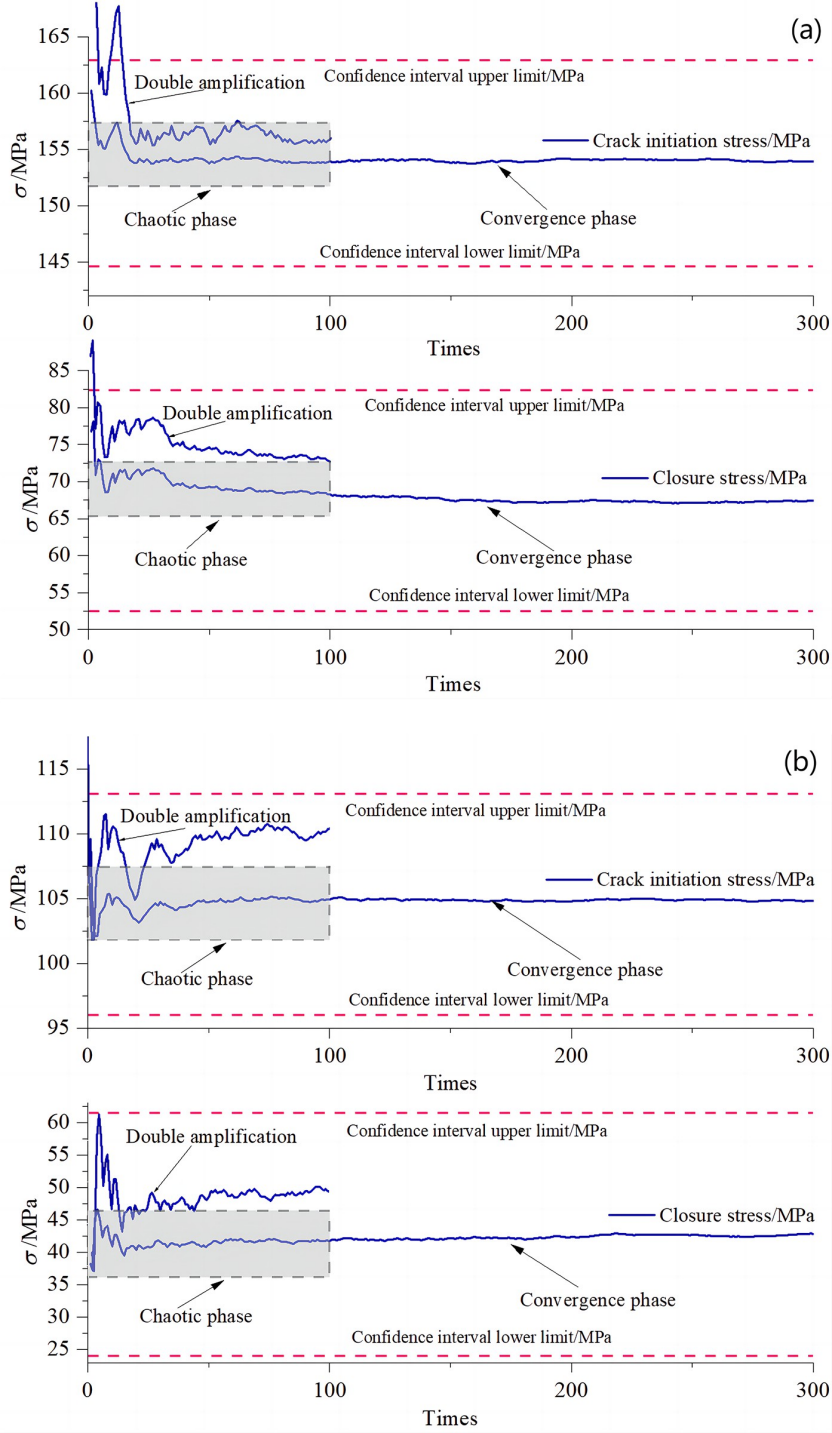
Monte Carlo simulation convergence process: (a) sample #3; (b) sample #4.

There was a large difference between the closure stresses and crack initiation stresses calculated by the 3 traditional analysis methods for the 5 groups of rock samples in the table. Therefore, it was difficult to determine whether the results calculated by any of the methods are accurate. In view of this, the results of the 3 methods were entered into the student *t* distribution Eq (6) to obtain reasonable intervals that satisfy their commonality. Since the 90% confidence interval balances accuracy and reliability, it was chosen for this paper [30]. As shown in Table 1, the confidence intervals calculated at a confidence level of 90% contain the maximum and minimum values for the three methods, and the size of the redundancy set for the confidence intervals varies according to the size of the variance between the methods. After determining the fuzzy first-stage stress interval, the second-stage convergence interval was further obtained using Monte Carlo probability sampling method within the interval according to Eq (7), and then the closure stress and crack initiation stress were calculated for each rock sample. The process is shown in Fig 8.

**Fig 9:**
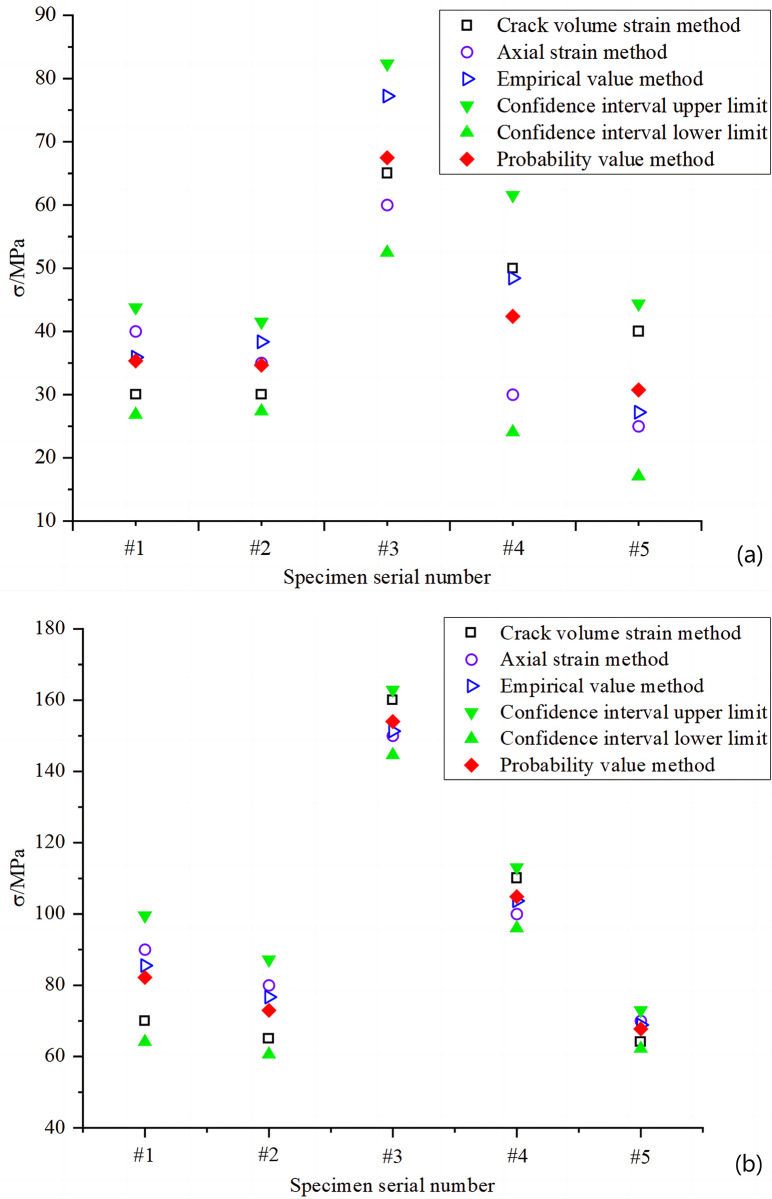
Comparison of results: (a) rock closure stress; (b) rock crack initiation stress.

It can be seen that the results after multiple probability sampling gradually approach the center of the student t-distribution. This indicates that they lie within the interval of the high confidence distribution common to multiple random sampling methods. And it is an excellent estimate that fully combines the advantages of the various methods. As can be seen in Fig 8, the 0–100 sampling process was very volatile. This chaotic phase can be seen as a transition from the first stage confidence interval to the second stage convergence interval. Convergence of samples began at around 100 random samples and essentially ended at around 300 samples. This convergence stage is considered to be in the second stage of the convergence interval. Therefore, this study took the average of 101–300 random samples in the convergence phase as the probability values of the closure stress and crack initiation stress of the rock. Thus, results combining the advantages of various methods are obtained. Comparison with the results of the 3 traditional methods is shown in Fig 9.

Analysis of the longitudinal comparison in Fig 9 shows that when determining the stress in the same rock sample, the different methods yielded very different results. For example, in determining the closure stress in Group #3 rocks, the difference between the empirical value method and the axial strain method is 27.8%. In addition, a comparative lateral analysis shows that there are large fluctuations when using the same method to determine the closure stress and crack initiation stress for different rock samples. For example, in Fig 9B, when the fracture volume method was used to determine the initial stresses for rocks 1, 2 and 5, the fracture volume method yielded the smallest values compared to the other methods. When applied to rocks 3 and 4, the crack volume method yielded the largest values compared to the other methods. The results of the probabilistic valuation method were analyzed by different methodological takes, which yielded results closer to the multi-value range. This is because the Student *t* distribution and Monte Carlo simulation methods restricted the results to high confidence intervals. For example, the result of the probabilistic valuation method to determine the closure stress for rock sample #5 is 30.74 MPa. This result is closer to most of the results of the empirical method (27.2 MPa) and the axial strain method (25 MPa). Subject to confidence intervals, the probabilistic valuation method has neutralized the detrimental effects of the extremes of different methods and reduced the occurrence of relative extremes leading to large errors. For example, the three traditional method closure stresses for rock sample #4 were 30 MPa, 50 MPa, and 48.3 MPa, with a standard deviation of 11. When the probabilistic valuation method was used, the standard deviation was reduced to about 9. Therefore, the probabilistic valuation method proposed in this paper combined the advantages of other methods with more accuracy and stability when applied to each rock sample.

## 4 Discussion

Currently, some studies are based on acoustic methods [31, 32] to determine the stress state of rocks faster. Traditional methods based on stress-strain curve analysis are slow and cumbersome, but are widely used because of their greater reliability. Therefore, this study used values from two methods of stress-strain curve analysis (axial strain method and crack volume method) and a modified version of these methods (empirical value method) as the base data in order to minimize the errors observed in determining the stage of rock damage. In addition, a growing number of studies have identified a variety of factors that influence the stresses in various stages of the rock. Examples include constant fatigue loading [33], water intrusion [34], initial crack volume [35], as well as temperature [36] and porosity [37]. Clearly, there is a need for combining similar probabilistic valuation methods with a variety of approaches to meet the need when considering the impact of multiple factors.

The student *t* distribution has heavier tails and better robustness. The larger the values in the tails at both ends, the higher the probability of extreme events compared to a normal distribution. In the case of the data set of this study, the two test methods (axial strain method and crack volume strain method) are at the two ends of the numerical interval. This means that obtaining values at both ends of the interval has a higher reliability [13]. In addition, better robustness in the student *t* distribution, which makes models more resistant to interference and better performance.

The use of Monte Carlo sampling method improved the accuracy of models. As the sample size increased, the stress values gradually converged from random variations. More sample size will bring the results of the model progressively closer to the true values.

Although this study has determined the closure and onset stresses in rocks by probabilistic methods, there are still some areas for improvement:

1. The accuracy of Monte Carlo is related to the number of samples. With larger amounts of data, an increase in the number of samples may increase model complexity. So how to balance the accuracy of the model results and the time of the sampling process is also an issue that needs to be addressed in the future.
2. With the popularity of computer technology in the field of rock mechanics, the application of numerical simulations [38] and intelligent algorithms [39] will be a hot topic in the future. Therefore, in the subsequent research, we consider embedding the probabilistic valuation method into numerical simulation software or for the optimization of intelligent algorithms. Thus, the errors of different methods will be reduced under various operating conditions. For example, a non-contact method (digital image correlation) is used in conjunction to identify the stress threshold of a rock. This method can overcome the vibration noise confusion due to the brittleness of the rock. Generating images using numerical simulations, followed by deep learning to identify the stress thresholds of rocks is also a major direction we need to focus on in the future.

## 5 Conclusion

In order to make an accurate and rapid judgement of the rock stress state, this study proposes a probabilistic valuation method for determining rock closure stress and initiation stress based on Student t distribution theory and Monte Carlo stochastic simulation theory. The conclusions are as follows:

1. Confidence intervals for the values of multiple rock stress determination methods were calculated using the student *t* distribution method, where the advantages of each method are fully combined.
2. Monte Carlo simulation was used to further reduce the confidence intervals, resulting in smaller and more accurate rock closure stress and crack initiation stress with less ambiguous properties.
3. Comparison of the results applied to 5 sets of rock samples with the values obtained by the other 3 methods showed that the probabilistic valuation method has better accuracy and stability.
